# A9 LOSS OF INTESTINAL EPITHELIAL NCOR1 INCREASES OXIDATIVE METABOLISM

**DOI:** 10.1093/jcag/gwad061.009

**Published:** 2024-02-14

**Authors:** M Lecours, A Di Castro, J Herrera Pulido, M Gendreau, C Jones, N Perreault, F Boudreau

**Affiliations:** Universite de Sherbrooke, Sherbrooke, QC, Canada; Universite de Sherbrooke, Sherbrooke, QC, Canada; Universite de Sherbrooke, Sherbrooke, QC, Canada; Universite de Sherbrooke, Sherbrooke, QC, Canada; Universite de Sherbrooke, Sherbrooke, QC, Canada; Universite de Sherbrooke, Sherbrooke, QC, Canada; Universite de Sherbrooke, Sherbrooke, QC, Canada

## Abstract

**Background:**

The nuclear co-repressor NCOR1 acts as a central platform of a complex that represses gene expression associated with inflammatory response. Literature also recently uncovered its possible role in oxidative metabolism in some tissues and organs. Although intestinal epithelial NCOR1 is crucial to restrict IBD symptoms during experimental colitis, its precise role in controlling intestinal epithelial cell biology has not been established.

**Aims:**

We aimed to investigate the role of intestinal epithelial NCOR1 in oxidative metabolism.

**Methods:**

Crossing Villin-Cre and *Ncor1*^loxP/loxP^ mice was performed to delete *Ncor1* in the whole intestinal epithelium conditionally. Crypts from control and NCOR1^ΔIEC^ littermates were isolated to generate colonoids 3D cultures. Phenotypic, genotypic, and transcriptomic analyses were performed on those colonoids.

**Results:**

Colonoids from NCOR1^ΔIEC^ mice were phenotypically smaller but were found to display drastic higher intestinal stem cell propagation features during organoid clonogenic assays. A Gene Set Enrichment Analysis from RNA sequencing predicted an increase in oxidative metabolism in NCOR1^ΔIEC^ colonoids. Several genes associated with oxidative metabolism were confirmed to be modulated in both NCOR1^ΔIEC^ colonoids and the colon of NCOR1^ΔIEC^ mice. Mitochondrial mass was also significantly increased in colonoids when NCOR1 was absent. Loss of the co-repressor did not affect the expression of PGC1-α, a known antagonist of NCOR1. However, transcripts of cytochrome c somatic (*Cycs*), a target gene of PGC1-α, were found higher in NCOR1^ΔIEC^ colonoids, suggesting that the coactivator activity is increased in the absence of NCOR1.

**Conclusions:**

Our results highlight a new role for NCOR1 in the oxidative metabolism of intestinal epithelial cells. Its action via PGC1-α activity could potentially be targeted as a therapeutic measure during inflammatory bowel diseases.

**Funding Agencies:**

CIHR

